# Dynamic prediction of outcome for patients with severe aortic stenosis: application of joint models for longitudinal and time-to-event data

**DOI:** 10.1186/s12872-015-0035-z

**Published:** 2015-05-07

**Authors:** Eleni-Rosalina Andrinopoulou, Dimitris Rizopoulos, Marcel L. Geleijnse, Emmanuel Lesaffre, Ad J. J. C. Bogers, Johanna J. M. Takkenberg

**Affiliations:** Department of Biostatistics, Erasmus MC, Rotterdam, The Netherlands; Department of Cardiology, Erasmus MC, Rotterdam, The Netherlands; KU Leuven, L-Biostat, Leuven, Belgium; Department of Cardiothoracic Surgery, Erasmus MC, Rotterdam, The Netherlands

**Keywords:** Valvular disease, Brain natriuretic peptide, Aortic valve disease, Survival, Individualized prediction

## Abstract

**Background:**

Physicians utilize different types of information to predict patient prognosis. For example: confronted with a new patient suffering from severe aortic stenosis (AS), the cardiologist considers not only the severity of the AS but also patient characteristics, medical history, and markers such as BNP. Intuitively, doctors adjust their prediction of prognosis over time, with the change in clinical status, aortic valve area and BNP at each outpatient clinic visit. With the help of novel statistical approaches to model outcomes, it is now possible to construct dynamic event prediction models, employing longitudinal data such as AVA and BNP, and mimicking the dynamic adjustment of prognosis as employed intuitively by cardiologists. We illustrate dynamic prediction of patient survival and freedom from intervention, using baseline patient characteristics and longitudinal BNP data that are becoming available over time, from a cohort of patients with severe aortic stenosis.

**Methods:**

A 3-step approach was employed: (1) construction of a mixed-effects model to describe temporal BNP progression, (2) jointly modeling the mixed-effects model with time-to-event data (death and freedom from intervention), and (3) using the joint model to build subject-specific prediction risk models. The dataset used for this purpose includes 191 patients with severe aortic stenosis who were followed over a 3-year time period.

**Results:**

In the mixed-effects model BNP was significantly influenced by time, baseline patient age, gender, LV fractional ejection fraction and creatinine. Additionally, the joint model showed that an increasing BNP trend over time was found to be a significant predictor of death.

**Conclusions:**

By jointly modeling longitudinal data with time-to-event outcomes it is possible to construct individualized dynamic event prediction models that renew over time with accumulating evidence. It provides a potentially valuable evidence-based tool for everyday use in medical practice.

## Background

In clinical practice, physicians utilize different sources of information to predict patient prognosis. For example, in diagnosing a new patient with severe aortic stenosis (AS), a cardiologist considers not only the severity of the AS (for example through aortic valve area AVA measurement) but also patient characteristics such as patient age and comorbidities, New York Heart Association (NYHA) functional class and patient history, in order to make an assessment of patient prognosis. Additionally, biomarkers such as brain natriuretic peptide (BNP) can be used to further assess AS severity and prognosis. A small AVA and a high BNP are both associated with a more severe disease and a worse outcome [1-3].

Empirically, cardiologists adjust their prognosis over time at each outpatient clinic visit, with the change in functional class, AVA and BNP. Based on emerging evidence on determinants of the outcome in AS, and with the help of novel statistical approaches to model outcomes, it is now possible to construct dynamic prediction models for patient outcome, employing repeatedly collected (longitudinal) data such as BNP, mimicking the dynamic adjustment of prognosis as employed intuitively by cardiologists at each outpatient clinic visit.

This paper aims to illustrate the use of joint models of longitudinal and time-to-event data to dynamically predict individualized event occurrence severe AS. For this purpose, data from a prospective cohort study of 191 patients with severe AS is modeled to dynamically predict prognosis of two patients: Mr. Jones and Mr. Smith; who were recently diagnosed with severe AS.

## Methods

### Patient dataset

We used the patient dataset of a previously reported prospective cohort study of 191 adult patients, who were diagnosed with severe aortic valve disease in seven cardiology clinics in the wider Rotterdam area between 2006 and 2009, and who were followed for 2 years [4]. Inclusion criteria were AVA ≤ 1 cm^2^, peak transaortic jet velocity (Vmax) ≥ 4 m/s, or aortic valve/left ventricular outflow tract velocity time integral ratio ≥ 4. The patients were followed clinically, including BNP measurements, and echocardiographically at baseline and then after 6, 12 and 24 months. Baseline patient characteristics are displayed in Table 1. In total 561 BNP measurements were collected over a 2-year period (mean 0.9 years; range 0–2.5 years). During the follow-up period, 15 % of the patients (N = 28) died and 48 % (N = 91) received an aortic valve replacement of transcatheter aortic valve implantation.

**Table 1:** Baseline patient characteristics

	All patients (Number = 191)
Male gender (n, %)	118, 62 %
Age in years (mean, sd)	72.6, 11.4
Symptomatic at study entry (n, %)	132, 69 %
Smoking (n, %)	115, 60 %
Hypertension (n, %)	100, 52 %
Diabetes (n, %)	39, 20 %
Dyslipidemia (n, %)	93, 49 %
AVA in cm^2^ (mean, sd)	0.74, 0.27
LV ejection fraction in % (mean, sd)	61, 6.7
Creatinine in micromol/L (mean, sd)	89, 125

AVA = aortic valve area; LV = left ventricular

The study protocol was approved by the medical ethics committee of Erasmus University Medical Center (MEC 2006–066); all patients provided written informed consent.

### Statistical methods

The development of a dynamic event prediction model that takes into account both baseline patient characteristics and longitudinal BNP measurement, requires that we first describe the evolution of BNP over time, correcting for baseline variables. Second, we use this information in a time-to-event model. Finally, using the combined model, we perform dynamic event predictions. In the next paragraphs we describe in detail the statistical methods that were employed in this 3-step process, and the rationale behind these methods.

First, we fitted a mixed-effects model to describe the evolution of BNP over time. Particularly, the model included time (years) and the baseline covariates: AVA (cm^2^), patient age (years), symptoms (yes/no), gender, transformed LV ejection fraction (%) and transformed creatinine (micromol/L). Transformation was done by dividing the values with the standard deviations of the specific covariates. Moreover, due to heterogeneity in the residuals plot the logarithmic scale of BNP was used. An advantage of the mixed-effects models is that they account for the positive correlation between the measurements that are observed within the same patient. For example, the values of BNP that are observed over time from the same patient are expected to be more correlated than between patients. Moreover, these models account for the biological variability in the longitudinal outcome. Specifically, if we measure BNP twice a day, we may not obtain the same result. By taking this into account using the mixed-effects model, more reliable results will be observed.

Second, to investigate the effect of the repeated BNP measurements on death and intervention probabilities, separate joint models of longitudinal and survival outcomes were constructed [5,6]. AVA, age, symptoms, gender, LV fraction and creatinine (all at baseline) were included as additional confounders. More details about the joint models are presented in the Appendix.

Third, we considered the joint modeling framework and focused on the assessment of the predictive ability of our survival outcomes. Specifically, it was of interest to predict patient survival and aortic valve intervention-free for a new patient that has provided us with a set of BNP measurements and baseline characteristics, using the fitted joint model for all patients. Due to the fact that BNP is time-dependent and not constant between the visits and therefore providing longitudinal measurement up to a specific time*,* assumes survival up to this time, it was more relevant to calculate the probability of surviving a future time point, given that the patient was alive until his last follow-up visit [7,8]. Using this approach, we applied the resulting joint modeling framework to two hypothetical patients: Mr. Jones and Mr. Smith and predicted their future survival and aortic valve intervention-free probabilities. Specifically, Mr. Jones is a 72 year old male, with creatinine value at baseline 92 micromol/L, AVA of 0.96 cm^2^, LV ejection fraction 61 % and BNP values over time 64, 70, 72 and 78 pg/ml measured at 0.5, 0.9, 1.5 and 1.5 years. Moreover, he is asymptomatic at baseline. Additionally, Mr. Smith is a 79 year old male that has creatinine equal to 92 micromol/L, AVA equal to 0.61 cm^2^, LV ejection fraction equal to 61 % and he is symptomatic at baseline. Finally, his BNP values are 381, 287, 1068 and 1070 pg/ml measured at 0, 0.9, 1.2 and 2 years.

Furthermore, we performed internal validation using a bootstrapping procedure (size of 1000). Specifically, we focused on discrimination, that is, how well can the model discriminate between patients who are about to experience the event within a time frame after the last measurement, from patients that are going to surpass this time frame. Since the patients were visiting their physician approximately every half year, we set this time frame. In particularly, we relied on the receiver operating characteristic (ROC) approach to assess the predictive ability of the longitudinal biomarker BNP [7].

All analyses have been implemented in R-3.2.0, which can be downloaded as freeware at http://www.r-project.org, using the JM package [9].

## Results

**Fig. 1:**
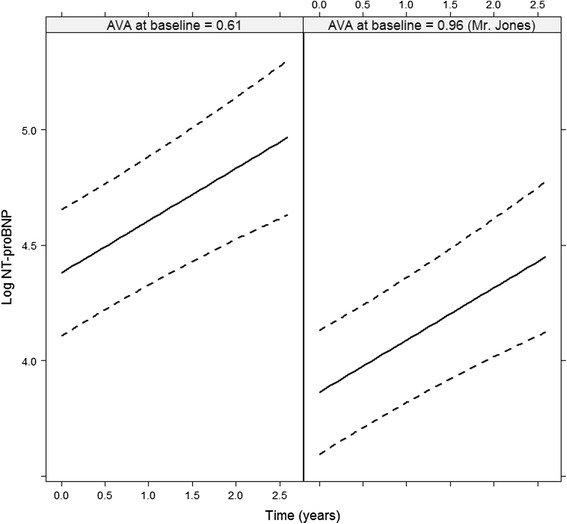
Effect plot of AVA described from the joint model for Mr. Jones and another patient with the same age, with impaired LV ejection fraction of 61, creatinine level equal to 92 and both patients with no symptoms at baseline

As illustrated in Table 2 in the mixed-effects model describing the evolution of BNP over time, all covariates have a strong association with the levels of BNP, except baseline gender. Specifically, a longer follow-up, lower AVA at baseline, older patient baseline age, symptomatic patient at baseline, lower baseline LV ejection fraction and a higher baseline serum creatinine are highly associated with an increased BNP. Moreover, Fig. 1 shows the evolutions of BNP over time of two hypothetical patients, Mr. Jones and another patient that has the same characteristics as Mr. Jones except for the AVA level which is 0.61. It is obvious in Fig. 1 that a smaller AVA is associated with a higher BNP at baseline. Furthermore, there is no difference in the progression of BNP between the two patients. From the joint model with the death as outcome, in Table 3, we observe that smaller AVA at baseline, male patient, symptoms at baseline and higher BNP at a specific time point (since we used all repeated measurements in the model for the specific covariate) tend to be associated with death. The joint model with the aortic valve intervention as outcome shows that a younger patient and symptoms at baseline are strongly associated with aortic valve intervention probabilities.

**Table 2:** Coefficients, standard error of coefficients and p-values for the mixed-effects model describing the evolution of BNP over time

	Coef	Se(coef)	p-value
(Intercept)	2.92	0.95	0.0025
Time (years)	0.23	0.04	<0.0001
AVA (cm^2^)	−1.48	0.3	<0.0001
Age (years)	0.05	0.007	<0.0001
Symptoms	0.43	0.18	0.0188
Male gender	−0.34	0.18	0.0607
*LV ejection fraction (%)	−0.16	0.08	0.0486
*Creatinine (micromol/L)	0.4	0.09	<0.0001

AVA = aortic valve area; LV = left ventricular. * Trasnformed LV ejection fraction and Creatinine in the models

**Table 3:** Coefficients, standard error of coefficients and p-values for the joint model predicting death and aortic valve intervention

	Coef	Exp(coef):HR	Se(coef)	p-value
*Death*				
BNP at specific time point (pg/ml)	0.5	1.65	0.3	0.0962
AVA (cm^2^)	−2.61	0.07	1.5	0.0815
Age (years)	0.02	1.02	0.04	0.5674
Male gender	1.12	3.06	0.6	0.0623
Symptoms	1.87	6.49	1.05	0.0753
*LV ejection fraction (%)	0.01	1.01	0.25	0.9539
*Creatinine (micromol/L)	0.18	1.2	0.15	0.2162
*Aortic valve intervention*				
BNP at specific time point (pg/ml)	0.18	1.2	0.25	0.4787
AVA (cm^2^)	−1.12	0.33	1.04	0.2804
Age (years)	−0.04	0.96	0.02	0.0077
Male gender	0.39	1.48	0.49	0.4287
Symptoms	1.08	2.94	0.46	0.0183
*LV ejection fraction (%)	0.24	1.27	0.21	0.2388
*Creatinine (micromol/L)	−1.43	0.24	1.31	0.2761

BNP = brain natriuretic peptide; AVA = aortic valve area; LV = left ventricular; HR = hazard ratio. * Trasnformed LV ejection fraction and Creatinine in the models

**Fig. 2:**
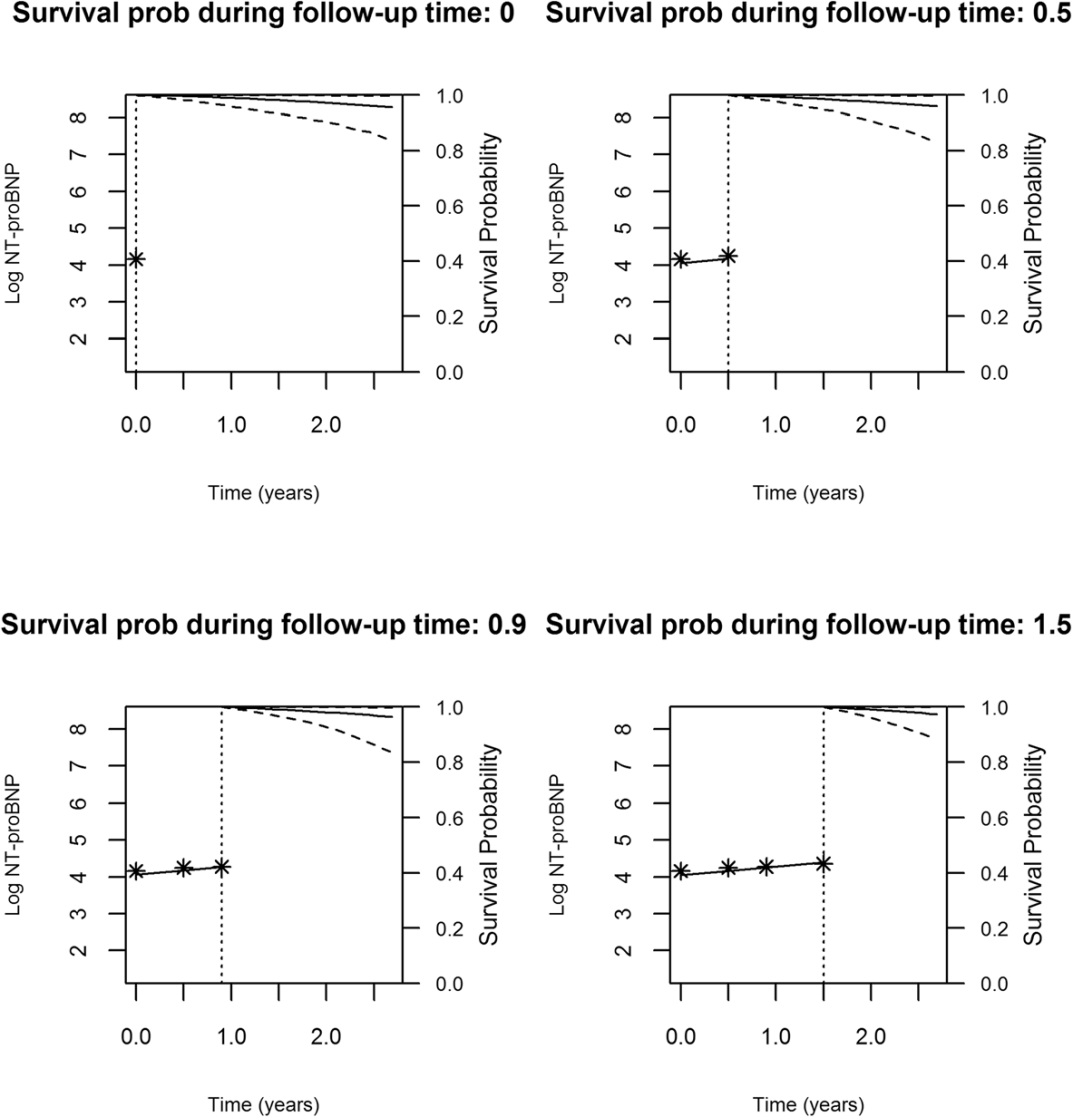
Dynamic prediction for the survival probability for Mr. Jones

**Fig. 3:**
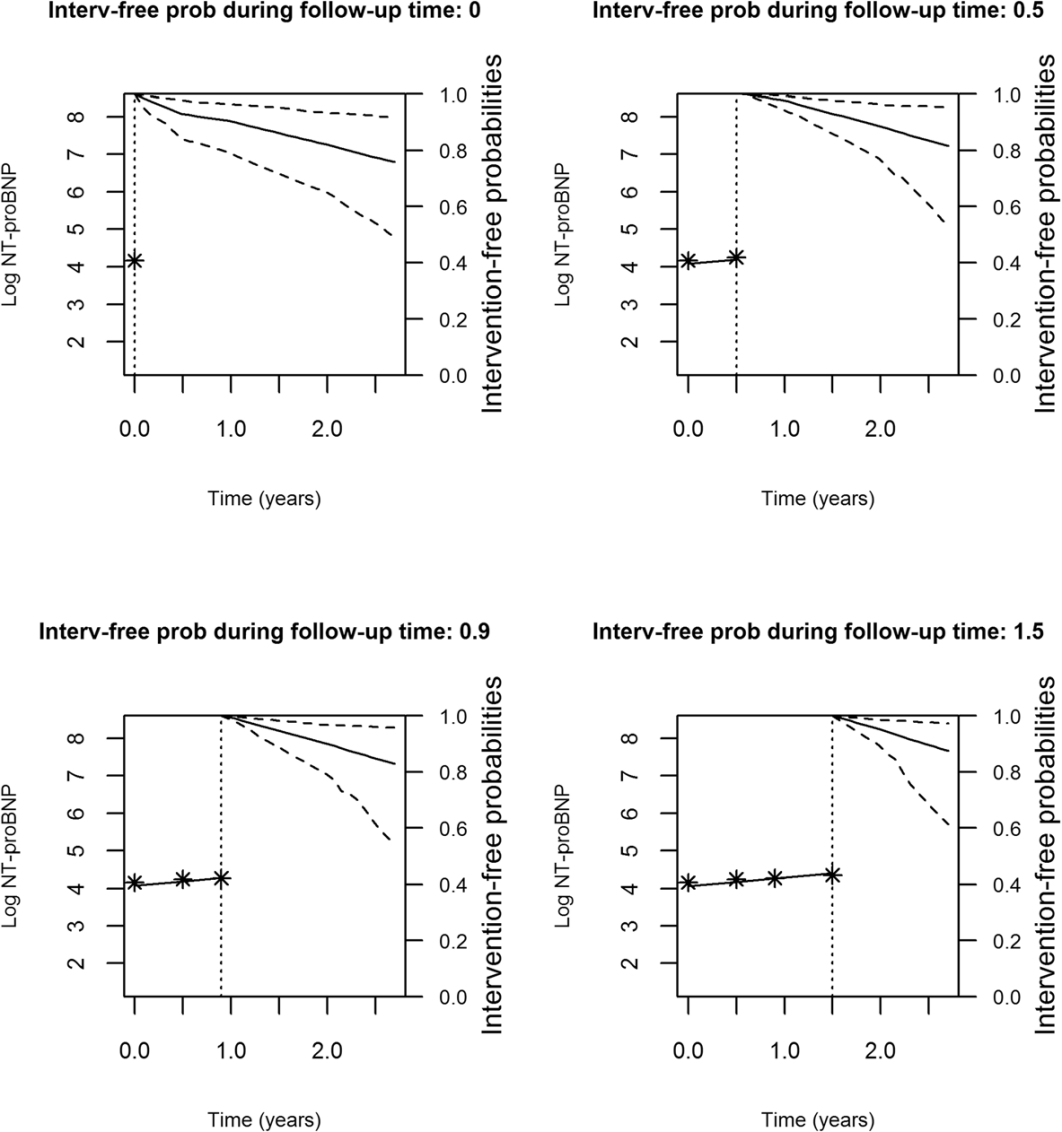
Dynamic prediction for the aortic valve intervention-free probability for Mr. Jones

**Fig. 4:**
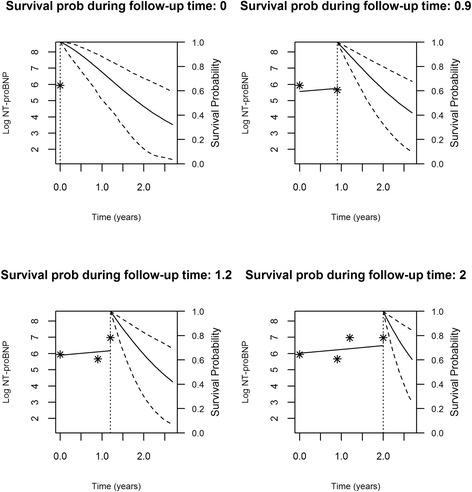
Dynamic prediction for the survival probability for Mr. Smith

**Fig. 5:**
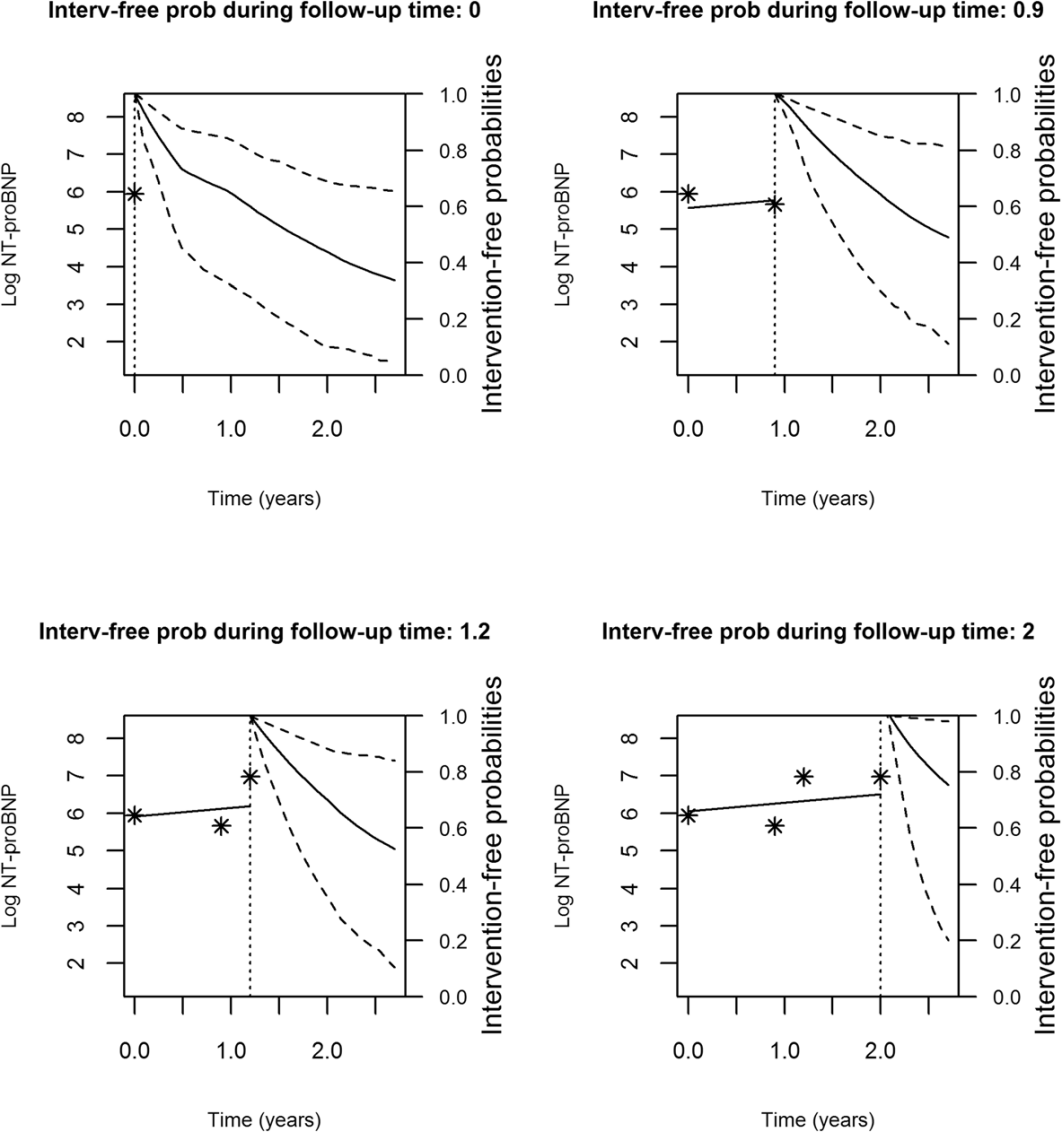
Dynamic prediction for the aortic valve intervention-free probability for Mr. Smith

Figures 2, 3, 4 and 5 represent the dynamic prediction of survival and aortic valve intervention-free respectively for Mr. Jones and Mr. Smith, employing the joint modeling framework. It can be seen in Fig. 2 that as more BNP measurements accumulated over time for Mr. Jones, the survival curve does not show big changes. Moreover, the same can be seen in Fig. 3, where the intervention-free probabilities are presented. This can be explained by the fact that Mr. Jones’ BNP measurements are relatively low and stable. In contrast, Mr. Smith has more steep curves for both expected survival and aortic valve intervention-free probabilities indicating that the patient should be monitored frequently. Specifically, one year after his first follow-up visit Mr. Smith has a survival probability of 70 %, while one year after his last visit his survival probability is less than 50 %. The reason could be that Mr. Smith has a high BNP value at baseline and his progression is faster within the 2 year period compared to Mr. Jones. Thus, Mr. Smith has a much lower survival probability one year after his last follow-up.

Finally, from the bootstrapping we observe the area under the ROC curve for death and aortic valve intervention to be 0.88 and 0.59, respectively. This indicates a good discriminative capability of the BNP for death, and little added value for the prediction of aortic valve intervention.

## Discussion

In this paper we illustrated the use of joint models of longitudinal and time-to-event data for individualized dynamic event prediction using serial BNP measurements in patients with severe AS. Patient prognostication may be improved by the use of such models that take into account all available medical information that accumulates over time. In the case of Mr. Jones and Mr. Smith, their probabilities of survival and aortic valve intervention-free were calculated accounting for all BNP values that accumulated over time and were updated when new BNP measurements became available. This approach provides the cardiologist with a useful evidence-based tool to assess the impact of BNP on patient prognosis. Importantly, the calculated probabilities for survival and aortic valve intervention-free can be used as an early warning system, allowing the necessary time for the physicians to plan an intervention. Given the impaired quality of life (QOL) of symptomatic patients with AS [10] and the considerable improvement in QOL after the aortic valve replacement, dynamic event predictions may be of great value in optimizing the timing of the intervention [11].

The joint model of longitudinal and survival data [5] represents a powerful statistical tool capable of capturing the association between longitudinal and survival data. An alternative approach is to utilize the time-dependent Cox model. However, this model assumes a step function between the repeated measurements, which is not realistic for biomarkers due to the fact that such cardio data as BNP values cannot be assumed to be constant between visits.

Of course, the proposed methodology has several (potential) limitations, both from a clinical and a statistical point of view. From a clinical point of view, every patient is unique, and analysis based on group data may not account for the special characteristics of an individual patient. Moreover, there are factors that are not included in the statistical models that may play an important role and thus influence the decision making. In this respect we acknowledge that the proposed methodology may be supportive in clinical decision making, but can never replace clinical expertise. Also, for clinicians with limited understanding of advanced statistical models, the proposed methodology may be difficult to comprehend, and tutorials aimed at clinicians are needed to further educate clinical professionals [6]. From the statistical point of view, the analysis of more than one longitudinal outcomes such as BNP, AVA and symptoms over time together with survival outcomes requires advanced computational work and standard statistical packages do not yet provide these options. Moreover, there is not yet a package performing dynamic event prediction accounting for the competing risk problem: specifically, patients could die or require an intervention, in this case is aortic valve intervention. The analysis, then, becomes more complicated by the fact that the two censored outcomes are not completely independent, thus it is clear that analyzing the two outcomes separately is not appropriate and may lead to bias. However, in order to keep the analysis simple and thus to use only available packages, in the paper we did not accounted for the competing risk problem. Furthermore, a topic that was not addressed in this paper, concerns the validation of the derived predictions in terms of calibration. Within the joint modeling frame, some work has been done by Rizopoulos (2011) [7] and Proust-Lima and Taylor (2009) [8]. Specifically, they focus on predictive accuracy measures that compare the actual value of predictions with the observed data using simulated data. Finally, a dataset consisting of more patients that are followed for a longer time period may provide better predictions for future patients.

Although all analysis was performed using standard statistical packages, a level of expertise in programming may be required. Thus, interactive web applications with friendly controls that easily incorporate plots and summaries are essential for adequate implementation of the proposed models in clinical practice may be interesting to produce. Particularly, an easy web application could give the opportunity to every physician to derive updated predictions for new patients when more longitudinal outcomes are available.

From the analysis we obtained a non-significant association between aortic valve intervention and the evolution of BNP (Table 3). Hence, the validation showed that for the target group of patients the BNP as a marker for intervention does not exhibit great discrimination power. BNP levels have been previously found to be predictors of reoperation. Therefore, although BNP profile is not a good predictor of intervention in our case, it is reliable in predicting mortality and thus can be very helpful in planning an intervention to prevent mortality due to AS disease progression. This non-significant result could be explained by the fact that additional cardiovascular risk factors were not taken into account because either there were not available or the patients were not enough to include more factors in the model.

In this paper we assumed linear trajectories for the BNP biomarker since we did not have a big range of values per patient. However, in a different setting where more information would be available per patient it may be of interest to investigate for non-linear profiles. Specifically, patients could have highly non-linear evolution that could not be described by a simple structure, such as linear one. Even though the interpretation then becomes more complex, it is evident that misspecification of the evolution of the biomarkers could lead to bias. In order to obtain valid results, it is important to postulate a mixed-effects model that is capable of appropriately capturing such non-linear evolutions.

## Conclusions

In conclusion, this paper has shown that temporal adjustment of risk prediction models for patients with severe AS, as more measurements of BNP become available over time, provide the physician with an evidence based understanding of the prognostic implication of changes in the patient’s disease condition. With the cardiovascular medical practice increasingly moving towards personalized medicine [12], joint models may provide an attractive tool for subject-specific predictions. The proposed joint model that was built and used to predict prognosis of patients suffering from severe AS, can be easily extended to other chronic disease entities that employ both longitudinal and survival data to dynamically assess patient prognosis.

## Appendix

*Introduction to joint models*

Let *T*_*i*_^*^ denote the true failure time for the *i*-th individual (*i* = 1,…, n), and *C*_*i*_ the censoring time, then *T*_*i*_ = min(*T*_*i*_^*^, *C*_*i*_) represents the observed failure time for the *i*-th patient. Moreover, *δ*_*i*_ = 0,1 is the event indicator where 0 indicates censoring. For the longitudinal part, we let ${y}_i$ consist of longitudinal responses that may be obtained at different time points *t*_*ij*_ and have length *n*_*i*_. To describe the subject-specific evolutions over time of the longitudinal outcome we utilize a linear mixed-effects model. Specifically, it takes the form,

$$ {y}_i(t)={f}_i(t)+{\mathit{\in}}_i={x}_i^T(t)\beta +{z}_i^T(t){b}_i+{\mathit{\in}}_i, $$

where *x*_*i*_(*t*) denotes the design vector for the fixed effects regression coefficients *β* and *z*_*i*_(*t*) the design vector for the random effects *b*_*i*_.

Finally, we assume that a normal distribution for the random effects describes the evolution of the longitudinal outcomes, i.e.,

$$ {b}_i\sim N\left(0,{\sigma}_b\right), $$

Where *σ*_*b*_ is the variance of the random intercept.

For the survival process we have

$$ {h}_i\left(t,{\theta}_s\right)={h}_0(t){\mathrm{e}}^{\left\{{\gamma}^T{\omega}_i+a{f}_i(t)\right\}}, $$

where *θ*_*s*_ is the parameter vector for the survival outcomes, *ω*_*i*_ is a vector of baseline covariates with a corresponding vector of regression coefficients *γ*, and *α* denotes the strength of association between the longitudinal and survival outcome. Moreover, a Weibull baseline hazard *h*_*0*_*(t) = ψt*^*ψ–1*^ was assumed.
